# Berberine attenuated pro-inflammatory factors and protect against neuronal damage via triggering oligodendrocyte autophagy in spinal cord injury

**DOI:** 10.18632/oncotarget.21203

**Published:** 2017-09-23

**Authors:** Hongyu Wang, Chang Liu, Xifan Mei, Yang Cao, Zhanpeng Guo, Yajiang Yuan, Ziming Zhao, Changwei Song, Yue Guo, Zhaoliang Shen

**Affiliations:** ^1^ Department of Orthopedics, First Affiliated Hospital of Jinzhou Medical University, Jinzhou City, PR China; ^2^ Department of Endocrinology, First Affiliated Hospital of Jinzhou Medical University, Jinzhou City, PR China; ^3^ Department of Stomatology, Second Affiliated Hospital of Jinzhou Medical University, Jinzhou City, PR China; ^4^ Department of Orthopedics, Second Hospital of Jinzhou City, Jinzhou City, PR China

**Keywords:** berberine, spinal cord injury, autophagy, oligodendrocyte, inflammation

## Abstract

Berberine exerts neuroprotective effect in neuroinflammation and neurodegeneration disease. However, berberine effect in acute spinal cord injury is yet to be elucidated. Herein, we investigated the neuroprotective effect of berberine in spinal cord injury (SCI). Sprague-Dawley rats were subjected to SCI by an intraperitoneal injection of berberine post-injury. The neurobehavioral recovery, cytokines of pro-inflammatory factors (TNF-α and IL-1β), autophagy-related proteins (LC3B, ATG16L, ATG7), and apoptosis-related protein cleaved caspase-3 were determined. The expressions of 2′, 3′-cyclic-nucleotide 3′-phosphodiesterase (CNPase), marker of oligodendrocyte, autophagy-related proteins ATG5 and neurons at the ventral horn were assessed. *In vitro*, the contents of the pro-inflammatory factors, TNF-α and IL-1β, were detected in the lipopolysaccharide (LPS)-treated primary spinal neuron. Berberine significantly improved the neurobehavior BBB score and attenuated the cytokines of pro-inflammatory factors in cerebrospinal fluid post-SCI. In addition, berberine upregulated CNPase positive oligodendrocyte expressing ATG5, promoted neuronal survival and reduced the cleaved caspase-3 expression after SCI. In primary spinal neuron, the LPS-induced inflammatory factors could be reduced by berberine, whereas the autophagy inhibitor, 3-Methyladenine reverses the effect. Berberine attenuated inflammation of the injured spinal cord and reduced the neuronal apoptosis via triggering oligodendrocyte autophagy in order to promote neuronal recovery.

## INTRODUCTION

Spinal cord injury (SCI) led to neuronal dysfunction, and worldwide, several individuals suffering from it typically has life-long consequences [1]. In the United States of America, 12000 new patients with spinal cord injury were added annually, and nearly $40.5 billion per year were cost to healthcare industry [2]. The pathological process of SCI follows primary and secondary injuries. Targeting the reduced secondary inflammation proved to promote neuronal recovery after SCI. The inflammatory response plays a critical role in a secondary injury that contributes to extending the neuronal cell death and dysfunction. The pro-inflammatory mediators including cytokines and chemokines were released as a cascade after SCI, which triggered secondary injury.

Berberine, an isoquinoline alkaloid, is a traditional Chinese medicine (TCM) used for the treatment of diarrhea for several centuries in history. Berberine exerted a variety of biochemical functions including the anti-inflammatory effect [3–7], lymphocytes immune regulation, cell cycle arrest, and regulation of cell survival in cerebral ischemia [8–13], amelioration of β-amyloid pathology, gliosis, and cognitive impairment in Alzheimer's disease or traumatic brain injury [14, 15]. However, in the neurodegeneration disease, berberine sensitized the neurons to glutamate injury in a concentration-dependent manner [16]. Berberine reduces the oxidative stress and vascular inflammation, suppresses atherogenesis [17], and inhibits glutamate release from cortical synaptosomes in nerve terminals [18]. Berberine also attenuated ischemia-induced apoptosis and inhibited the reactive astrogliosis and microglia activation [19].

Autophagy is a process, in which protein aggregates and damaged organelles are removed for maintaining the intracellular homeostasis during various cell stresses [20]. Our previous study showed that autophagy activation was beneficial for the neuronal recovery of SCI [21–23]; it promotes inflammatory clearance and cell resistance to ischemia or hypoxia. In addition, berberine induces autophagy in glioblastoma by targeting the AMPK/mTOR/ULK1-pathway [24]. However, another report showed that Berberine inhibited EV71 replication by downregulating autophagy and MEK/ERK signaling pathway [25]. The effect of Berberine on autophagy may be cell type-dependent. While berberine activated the autophagy flux in neuron and reduced cell apoptosis [26], the effect on SCI is yet to be elucidated.

In the current study, we found that berberine attenuated pro-inflammatory factors, TNF-α and IL-1β in SCI and triggered the oligodendrocyte autophagy-related proteins, LC3B, ATG7, and ATG16L and reduced neuronal apoptosis. Berberine may exert a neuroprotective effect in SCI by attenuating neuronal inflammation and promoting oligodendrocyte autophagy to effectuate neuronal recovery.

## RESULTS

### Berberine promotes neurobehavioral recovery by improving BBB score after SCI

**Figure 1:**
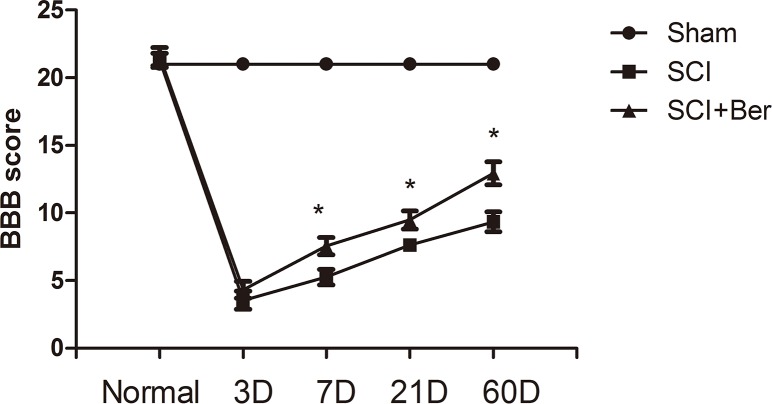
Berberine promotes neurobehavial BBB score at 7 days, 21 days, 60 days after SCI Berberine improves BBB score compare with SCI vehicle group at 7 days, 21 days, 60 days. ^*^P < 0.05 Berberine group vs SCI group. n=20

In order to explore the effects of berberine on the neuronal behavior recovery, we assessed the BBB score after SCI and found that normal rats gained a score of 21. Conversely, at 3 days after SCI, the scores of both berberine treatment and SCI groups were decreased and showed no significant difference after SCI. However, at 7 days after SCI, we found that the berberine treatment group showed a higher BBB score than the SCI vehicle group. Berberine improved the BBB score at both 21 and 60 days post-SCI than the SCI vehicle group (Figure 1).

### Berberine attenuated the pro-inflammatory factors TNF-α and IL-1β in the cerebral fluids after SCI

**Figure 2:**
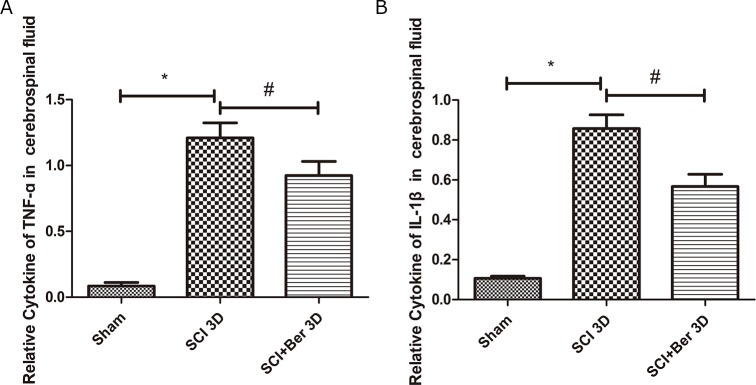
Berberine reduced cytokines of TNF-a and IL-1β at 3 days after SCI in the cerebrospinal fluid **(A)** Relative cytokines of TNF-a in cerebral fluids; **(B)** Relative cytokines of IL-1β in the cerebrospinal fluids. ^*^P < 0.05, SCI group vs sham group, ^#^ P < 0.05, Berberine group vs SCI group. n=20

To explore the effect of berberine on pro-inflammatory factors, we determined the levels of cytokines, TNF-α and IL-1β in the cerebral fluids post-SCI by ELISA. A significant increase was found in the cytokines, TNF-α and IL-1β, at 3 days after SCI, that was attenuated by berberine (Figure 2A, 2B).

### Berberine triggered the autophagy-related proteins, LC3B, ATG7, ATG16L, expression and reduced the apoptosis-related protein cleaved caspase-3

**Figure 3:**
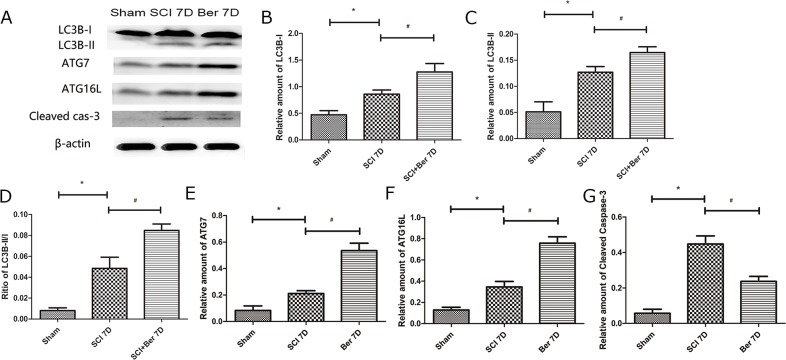
Berberine upregulated autophagy related protein LC3B-I/II, ATG7, ATG16L and Cleaved caspase-3 at 7 days after SCI **(A)** western blot of protein LC3B-I/II, ATG7, ATG16L and Cleaved caspase-3 at 7 days after SCI; **(B)** Relative amount of LC3B-I; **(C)** Relative amount of LC3B-II; **(D)** Ratio of LC3B-II/I; **(E)** Relative amount of ATG7; **(F)** Relative amount of ATG16L; **(G)** Relative amount of Cleaved caspase-3. ^*^P < 0.05, SCI group vs sham group, ^#^ P < 0.05, Berberine group vs SCI group. n=20

In order to explore the effect of berberine on spinal cord autophagy activity, we determined the expression of autophagy-related proteins, LC3B, ATG7, and ATG16L. We found that the autophagy-related proteins, LC3B-I/II, ATG7, and ATG16L were upregulated at 7 days post-SCI. However, berberine further promotes the expression of autophagy-related proteins, LC3B, ATG7, and ATG16L, suggesting an enhanced autophagy function by berberine (Figure 3A-3F). Furthermore, we observed the apoptosis-related protein cleaved caspase-3 and found that it was significantly increased after SCI, whereas berberine reduced the expression at 7 days post-SCI (Figure 3A, 3G). This phenomenon suggested a reduced neuronal apoptosis by berberine treatment.

### Berberine promotes CNPase-positive oligodendrocyte-expressing autophagy-related protein, ATG5, after SCI via IF staining

**Figure 4:**
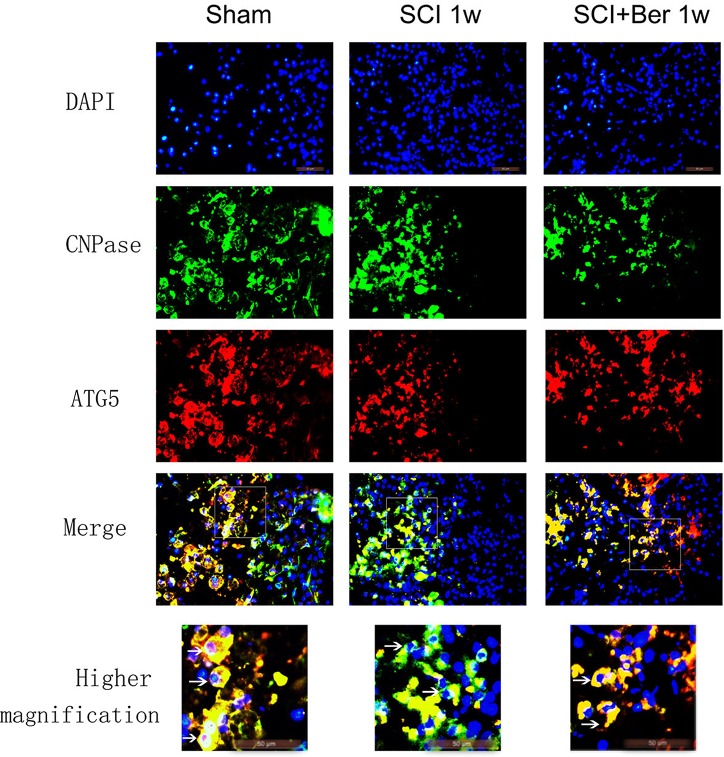
Berberine promotes CNPase/ATG5 double positive cells at 1 week after SCI Figures with the blue fluorescence dye stand for nucleus. Figures with the green fluorescence stand for CNPase positive cells. Figures with red fluorescence stand for ATG5 positive cells. Figures merged with DAPI/CNPase/ATG5 showed double-positive cells. White arrow showed positive expression cells (×1000). Scar bar was 50um.

**Figure 5:**
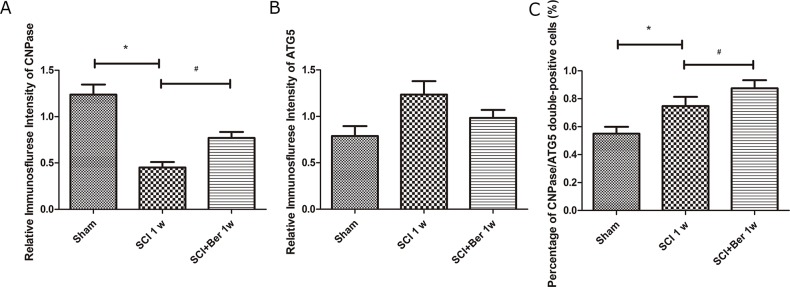
Berberine enhanced percentage of CNPase positive oligodendrocyte expressing ATG5 at 1 week after SCI **(A)** Relative immunofluorescence intensity of CNPase positive cells; **(B)** Relative immunofluorescence intensity of ATG5 positive cells; **(C)** percentage of CNPase/ATG5 double positive cells. ^*^P < 0.05, SCI group vs sham group, ^#^ P < 0.05, Berberine group vs SCI group. n=20

In order to investigate whether the increased autophagy was expressed in oligodendrocyte in SCI, the expressions of the oligodendrocyte marker CNPase and autophagy-related protein, ATG5, were examined by IF. We found that CNPase was highly expressed in the sham group, whereas the expression was significantly reduced post-SCI. Berberine treatment showed a higher CNPase expression than the SCI vehicle group, and the autophagy-related protein, ATG5s was expressed before and after SCI (Figure 4). Moreover, the quantitative analysis showed that berberine promotes the relative expression of CNPase after SCI and promotes a number of CNPase and ATG5 double positive cells (Figure 5A-5C).

### Berberine reduced cytokines, TNF-α and IL-1β, induced by LPS, and the effect was reversed by 3-MA, the autophagy inhibitor

**Figure 6:**
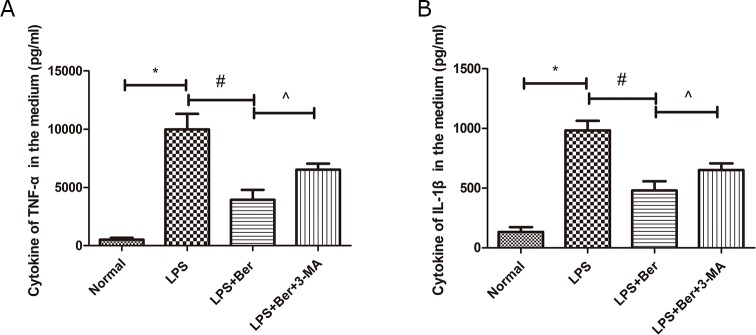
Berberine reduced cytokines of TNF-a and IL-1β in primary spinal neuron, while its effect was partly reversed by 3-MA, an inhibitor of autophagy **(A)** Cytokines of TNF-a in the medium; **(B)** Cytokines of IL-1β in the medium. ^*^P < 0.05, LPS group vs normal group, ^#^ P < 0.05, LPS+Ber group vs LPS group, ^P<0.05, LPS+Ber+3-MA group vs LPS+Ber group.

We collected the media of primary spinal neurons and found that the pro-inflammatory factors, TNF-α and IL-1β, were significantly increased after LPS treatment. However, berberine administration significantly reduced the levels of pro-inflammatory factors, whereas 3-MA, an inhibitor of autophagy, reversed the effect on these cytokines (Figure 6A, 6B).

### Berberine promotes neuronal survival at ventral horn of gray matter via nissl staining

**Figure 7:**
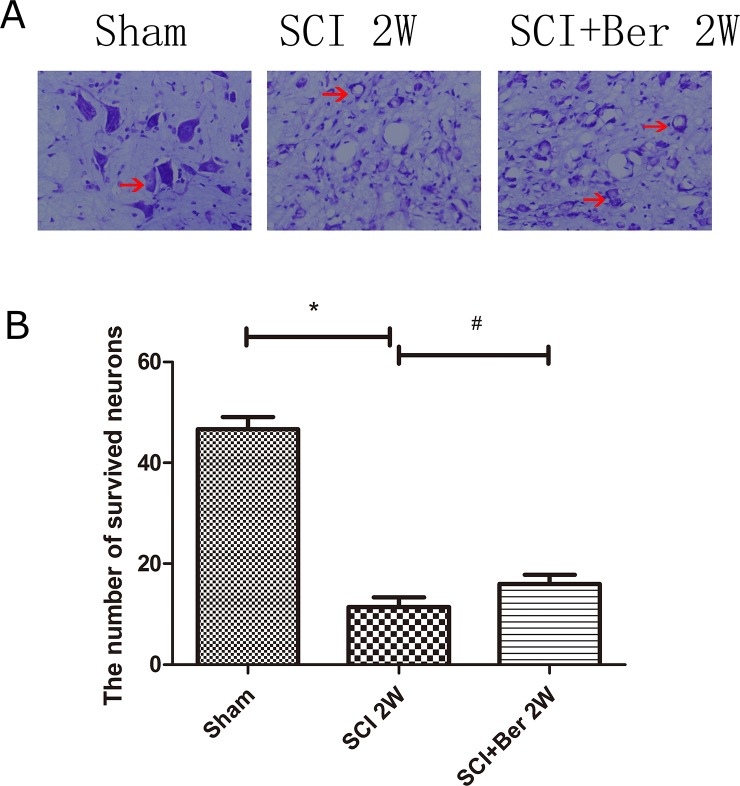
Berberine improved number of neurons at ventral horn of the spinal cord at 2 weeks via Nissl staining **(A)** Figures with red arrow are neurons (×400). Scar bar was 100um. **(B)** Quantitative analysis of survival neurons number at 2 weeks after SCI. ^*^P < 0.05, SCI group vs sham group, ^#^ P < 0.05, Berberine group vs SCI group. n=20

Nissl bodies were observed near the epicenter of the injury, demonstrating that the neuronal cells were significantly lost from the ventral horn post-injury, whereas, berberine promoted the survival of Nissl body-positive neurons at the anterior horn of the gray matter (Figure 7A). A quantitative analysis of the number of survived neurons showed that berberine enhanced the neurons’ survival at the ventral horn of gray matter post-SCI (Figure 7B).

## DISCUSSION

Berberine possesses various biological functions including anti-inflammation, anti-cancer [27], and cardiovascular protection [28, 29]. Previous studies proved a neuroprotective effect of berberine in neuronal degeneration and CNS injury [14, 15, 30]. The present study showed that berberine improved the neurobehavioral BBB score post-SCI. Berberine treatment attenuated the pro-inflammatory factors in the cerebral fluids as a consequence of SCI and reduced the LPS-induced inflammation that can be reversed by 3-MA, an autophagy inhibitor in primary spinal neuron culture. Furthermore, berberine promoted the expression of autophagy-related proteins, LC3B-I/II, ATG7, and ATG16L as assessed by Western blot, while that of the apoptosis-related protein cleaved caspase-3 was reduced at 7 days post-SCI. Our results showed that berberine promoted the number of CNPase-positive oligodendrocyte cells expressing the autophagy-related protein, ATG5, by IF staining. Nissl staining revealed a high number of surviving neurons at the ventral horn of gray matter as a result of berberine treatment, suggesting improved neuronal recovery. In addition, we demonstrate that berberine protected the neuronal survival via regulating the oligodendrocyte autophagy in order to accelerate the clearance of inflammatory factors and improve the neurobehavioral recovery after SCI.

Autophagy exerts a positive effect on CNS injury by removing the damaged mitochondria, scavenging the abnormally aggregated proteins, and compensating the energy deprivation [31]. In eukaryotes, the autophagic vesicles require a pair of essential ubiquitin-like conjugation systems, Atg12-Atg5 and Atg8 (LC3)-phosphatidylethanolamine (LC3-PE) [32]. Atg16L provides a functional link between the two critical ubiquitin-like conjugation systems of autophagy and binds with the Atg5 complex to pre-autophagosomal membranes, where it determines the site of LC3 lipidation and catalyzes the reaction essential for the formation of mature autophagosomes [33, 34]. Autophagy is one of the leading characteristics of tolerance against nutrient deprivation, such as ischemia or auto-inflammation. Berberine influenced the neuronal morphology by decreasing the hypoxia-induced apoptosis and promoting the LC3-dependent autophagy [26]. In addition, berberine attenuated the LPS toxicity to endothelial cells via JNK signaling [35]. The present study showed that berberine treatment promoted the expression of autophagy-related proteins LC3B, ATG7, and ATG16L and reduced that of the apoptosis-related protein, cleaved caspase-3 at 7 days post-injury, thereby suggesting that berberine may be involved in regulating autophagy and apoptosis in the process of SCI (Figure 3A-3G).

Berberine attenuated the inflammatory infiltration and reduced the permeability of the blood-brain barrier in autoimmune encephalomyelitis in C57BL/6 mice [36]. Berberine inhibited the palmitate-induced NLRP3 inflammasomal activation by triggering the autophagy in macrophages [37]. Also, it suppressed the activation of NLRP3 inflammasome and alleviated the MSU crystal-induced inflammationin rats [38]. Berberine inhibited the LPS-induced inflammation in microglia cells and protected the neuronal survival by reducing the inflammation in CNS injury [14, 29]. Our results showed that berberine reduced the cytokines of pro-inflammatory factors, TNF-α and IL-1β, in the cerebral fluids post-SCI (Figure 2A, 2B). The primary spinal neuron culture demonstrated that berberine reduced the levels of LPS-induced cytokines, TNF-α and IL-1β, whereas 3-MA reversed the anti-inflammatory effect (Figure 6A, 6B). This characteristic suggested that the anti-inflammatory effect of berberine may be partially correlated with its role in the regulation of autophagy.

Berberine exhibited its neuroprotective effect on oxygen-glucose deprivation/reperfusion in oligodendrocyte cells [26] against ischemia-induced excitotoxicity [39]. We found that as a result of SCI, the CNPase-positive oligodendrocyte cells were frequently co-expressed with autophagy-related protein, ATG5, and berberine improved the number of CNPase and ATG5 double positive cells (Figure 4 and 5A-5C). This phenomenon suggested that berberine may be involved in regulating oligodendrocyte autophagy to accelerate the clearance of injured organelles or excessive inflammation in SCI.

Berberine exerts a neuroprotective effect in ischemia in hippocampal cultures [40] and promotes neurite extension and axonal regeneration in sciatic nerves injury [41]. In our study, berberine showed a neuroprotective effect on neuronal survival at the ventral horn of gray matter via Nissl staining and berberine improved the neurobehavioral score by BBB test (Figure 1 and 7A-7B). Berberine promotes the neuronal recovery post-SCI.

The current study showed the neuroprotective effect of berberine in SCI. Berberine attenuated the pro-inflammatory factors, TNF-α and IL-1β, in the cerebral fluids after SCI. Berberine reduced the levels of cytokines, TNF-α and IL-1β, induced by LPS in the primary spinal neuron, while the effect can be partially reversed by the autophagy inhibitor, 3-MA. Berberine upregulated the oligodendrocyte autophagy level after SCI, promoted the survival of neuron at the ventral horn of gray matter, and reduced the neuronal apoptosis after SCI. The berberine effect of attenuating the neuroinflammatory factors might be partially correlated with its role in regulating the oligodendrocyte autophagy and accelerating locomotor recovery after SCI.

## MATERIALS AND METHODS

### Animals

Sixty adult female Sprague–Dawley (SD) rats (6-7 weeks of age, weighing 200–250g) were obtained from the Experimental Animal Center of Jinzhou Medical University. All experimental protocols were approved by the Animal Research Committee at First Affiliated Hospital of Jinzhou Medical University and conducted according to the Guide for the Care and Use of Laboratory Animals published by the US National Institutes of Health (NIH Publication No. 85–23, revised 1996). The results of these studies were reported according to the ARRIVE guidelines for experiments involving animal models [42].

### SCI model

Animals were subjected to spinal cord compression as described previously [21, 23, 43]. Briefly, the rats were anesthetized using 10%chloral hydrate (0.3 mL/kg for each rat), an incision was made at the dorsal midline, followed by cutting the subcutaneous layer and separating the muscle. A laminectomy was conducted at the T9-10 segment. After the spinal cord had been exposed, a weight-drop device weighing 10 g was dropped from a height of 25 mm and impacted on the spinal cord in the T9-10 segment. The contusion site of the spinal cord was significantly swollen and hemorrhaged accompanied by the posterior limb twitching rigidity. The muscular and subcutaneous skin was sutured under sterile conditions; the body temperature was monitored throughout the surgery by using a rectal probe, and the temperature was maintained at 37.0±0.5°C using a heated pad. The animals were housed in a specific pathogen-free laboratory with a standard 12 h light/dark condition at 25–30°C with food and water ad libitum. The rats were paralyzed immediately and moved sluggishly. The manual expression of the bladder was performed three times daily until the bladder function of the rats was reestablished. During experiment, 6 rats were sacrificed owing to the complications of intestinal obstruction post-SCI, and another 6 rats were supplemented in the deficient group.

### Drug administration

All 60 rats were randomly divided into three groups:

Sham group (N=20): rats received laminectomy alone;

SCI vehicle group (N=20): rats received SCI and equal volume of vehicle (0.9% saline);

SCI + berberine group (N=20): rats received SCI, and intraperitoneal injection of 3 mL berberine (10 mg/kg; Abcam) solubilized in 0.9% saline.

Drugs were administered intraperitoneally 10 min following injuryand one time every day for three days, consecutively, post-SCI. All measurements described below were also performed in a blinded manner.

The primary spinal neuron culture was stimulated by LPS (100 ng/mL, Sigma-Aldrich, St. Louis, MO, USA) for 12h, followed by berberine (1 μM, Abcam, Cambridge, UK) that was solubilized and diluted in the basal medium or 3-Methyladenine (3-MA; 5 μM, Sigma-Aldrich, St. Louis, MO, USA) for 12 h. The cells or supernatants in the medium were collected and used for subsequent experiments.

### Western blot

The protein was extracted from either T9-10 injured epicenter or uninjured control T9-10 spinal cord segments (5-6 mm) in phosphate-buffered saline (PBS)-perfused rats as described previously [44]. Briefly, an equivalent of 20 μg samples was loaded per well. The membranes were probed with rabbit polyclonal anti-LC3B [rabbit IgG, 1:1000; Cell Signaling Technology (CST), Danvers, MA], anti-ATG16L (rabbit IgG, 1:1000; CST), anti-ATG7 (rabbit IgG, 1:1000; CST), anti-cleaved caspase-3 (rabbit IgG, 1:1000; Abcam), and β-actin (rabbit IgG, 1:2000; Santa Cruz Biotechnology, Santa Cruz, CA, USA), respectively. The immunoreactive bands were visualized by an ECL detection system (Pierce Chemical, Rockford, IL, USA) and quantified by Image J software.

### Immunofluorescence (IF) staining

The animals were anesthetized and perfused with 4% ice-cold paraformaldehyde phosphate buffer (pH 7.4). Then, a 2-cm spinal cord segment around the epicenter was removed, and 10 μm sections were collected. Then, the sections were incubated with blocking buffer containing 0.01 M PBS, 0.1% Triton X-100, and 1% bovine serum albumin (BSA) for 30 min, followed by primary antibodies in blocking buffer at 4°C overnight in a humid chamber. The primary antibodies were anti-CNPase (2′, 3′-cyclic-nucleotide 3′-phosphodiesterase) (rabbit IgG, 1:200; CST) and anti-ATG5 (rabbit IgG, 1:400; CST). After washing with PBS for 3×10 min, the sections were incubated with Alexa Fluor 488/568 FITC rabbit anti-mouse secondary antibody at 1: 400 for 2 h at room temperature. The nuclei were counterstained using DAPI at a dilution of 1:1000 for 15 min. After three washes with PBS, the sections were covered with 50% glycerin. Images were captured on Leica DMI4000B microscope.

### Locomotion recovery assessment

The Basso, Beattie, and Bresnahan (BBB) open-field locomotor rating scale was employed for the evaluation of the recovery condition of motor function post-SCI. Briefly, three examiners independently assessed the BBB scores in a blinded manner before the operation and at 3, 7, 21, and 60 days after SCI. The BBB scores ranged from 0–21 points. The minimum points (0) indicated complete paralysis, and the maximum points implied normal function. The average scores were calculated according to the progress in locomotion recovery after SCI.

### ELISA

At 3 days post-SCI, the cerebral fluids were collected, and after 12 h culture of the primary spinal neuron, the conditioned medium was collected. A commercial ELISA kit (Abcam) estimated the levels of IL-1β and TNF-α according to the manufacturer's instructions. The data were analyzed by a microplate reader (Dynex Technology Chantilly, VA, USA).

### Nissl staining

10 μm-thick spinal cord sections of rats were prepared as previously described [45]. Brief, the sections were collected and stained using crystal violet in each group and differentiated by 95% and 100% alcohol, followed by a xylene rinse. The sections were observed by microscope and neurons counted randomly in five fields from the ventral horn of gray matter.

### Statistical analysis

Data were expressed as mean ± SD and analyzed using statistical analysis software SPSS, (version 17.0). The data were evaluated using Student's t-test for two groups and one-way analysis of variance (ANOVA) for more than two groups. P-values <0.05 was considered statistically significant.

## Abbreviations

BBB: Basso, Beattie, and Bresnahan
TNF-a: Tumor necrosis factor-alpha
IL-1β: Interleukin 1 beta
ELISA: enzyme-linked immunosorbent assay
LC3B: Light Chain 3
ATG16L: autophagy-related genes Atg16L1
ATG7: autophagy-related genes 7
ATG5: autophagy-related genes 5
CNPase: 2′, 3′-cyclic nucleotide 3′- phosphodiesterase
IF: immunoflurescence
LPS: Lipopolysaccharide
3-MA: 3-Methyladenine
SCI: Spinal cord injury
AMPK: AMP-activated protein kinase
Mtor: mammalian target of rapamycin
ULK1: unc-51 like autophagy activating kinase 1
EV71: enterovirus 71
ERK: extracellular regulated protein kinases
SD: Sprague–Dawley
PBS: phosphate buffered saline
BSA: bovine serum albumin
CNS: central nervous system
JNK: JunN-terminalkinase
NLRP3: nucleotide-binding oligomerization domain-like receptor pyrin domain containing 3
MSU: monosodium urate

## References

[1] Friedli L, Rosenzweig ES, Barraud Q, Schubert M, Dominici N, Awai L, Nielson JL, Musienko P, Nout-Lomas Y, Zhong H, Zdunowski S, Roy RR, Strand SC (2015). Pronounced species divergence in corticospinal tract reorganization and functional recovery after lateralized spinal cord injury favors primates. Sci Transl Med.

[2] SpinalCord Injury Facts and Figures at a Glance.

[3] Kulkarni SK, Dhir A (2010). Berberine: a plant alkaloid with therapeutic potential for central nervous system disorders. Phytother Res.

[4] Lu DY, Tang CH, Chen YH, Wei IH (2010). Berberine suppresses neuroinflammatory responses through AMP-activated protein kinase activation in BV-2 microglia. J Cell Biochem.

[5] Jia L, Liu J, Song Z, Pan X, Chen L, Cui X, Wang M (2012). Berberine suppresses amyloid-beta-induced inflammatory response in microglia by inhibiting nuclear factor-kappaB and mitogen-activated protein kinase signalling pathways. J Pharm Pharmacol.

[6] Zhang Q, Piao XL, Piao XS, Lu T, Wang D, Kim SW (2011). Preventive effect of Coptis chinensis and berberine on intestinal injury in rats challenged with lipopolysaccharides. Food and Chemical Toxicology.

[7] Li HM, Wang YY, Wang HD, Cao WJ, Yu XH, Lu DX, Qi RB, Hu CF, Yan YX (2011). Berberine protects against lipopolysaccharide-induced intestinal injury in mice via alpha 2 adrenoceptor-independent mechanisms. Acta Pharmacol Sin.

[8] Song B, Tang X, Wang X, Huang X, Ye Y, Lu X, Wei X, Zeng Y (2012). Bererine induces peripheral lymphocytes immune regulations to realize its neuroprotective effects in the cerebral ischemia/reperfusion mice. Cell Immunol.

[9] Chai YS, Hu J, Lei F, Wang YG, Yuan ZY, Lu X, Wang XP, Du F, Zhang D, Xing DM, Du LJ (2013). Effect of berberine on cell cycle arrest and cell survival during cerebral ischemia and reperfusion and correlations with p53/cyclin D1 and PI3K/Akt. Eur J Pharmacol.

[10] Zhou XQ, Zeng XN, Kong H, Sun XL (2008). Neuroprotective effects of berberine on stroke models in vitro and in vivo. Neurosci Lett.

[11] Zhang X, Zhang X, Wang C, Li Y, Dong L, Cui L, Wang L, Liu Z, Qiao H, Zhu C, Xing Y, Cao X, Ji Y, Zhao K (2012). Neuroprotection of early and short-time applying berberine in the acute phase of cerebral ischemia: up-regulated pAkt, pGSK and pCREB, down-regulated NF-κB expression, ameliorated BBB permeability. Brain Res.

[12] Hu J, Chai Y, Wang Y, Kheir MM, Li H, Yuan Z, Wan H, Xing D, Lei F, Du L (2012). PI3K p55γ promoter activity enhancement is involved in the anti-apoptotic effect of berberine against cerebral ischemia-reperfusion. Eur J Pharmacol.

[13] Durairajan SS, Liu LF, Lu JH, Chen LL, Yuan Q, Chung SK, Huang L, Li XS, Huang JD, Li M (2012). Berberine ameliorates β-amyloid pathology, gliosis, and cognitive impairment in an Alzheimer's disease transgenic mouse model. Neurobiol Aging.

[14] Chen CC, Hung TH, Lee CY, Wang LF, Wu CH, Ke CH, Chen SF (2014). Berberine protects against neuronal damage via suppression of glia-mediated inflammation in traumatic brain injury. PLoS One.

[15] Cai Z, Wang C, Yang W (2016). Role of berberine in Alzheimer's disease. Neuropsychiatr Dis Treat.

[16] Shin KS, Choi HS, Zhao TT, Suh KH, Kwon IH, Choi SO, Lee MK (2013). Neurotoxic effects of berberine on long-term L-DOPA administration in 6-hydroxydopamine-lesioned rat model of Parkinson's disease. Arch Pharm Res.

[17] Wang Q, Zhang M, Liang B, Shirwany N, Zhu Y, Zou MH (2011). Activation of AMP-activated protein kinase is required for berberine-induced reduction of atherosclerosis in mice: the role of uncoupling protein 2. PLoS One.

[18] Lin TY, Lin YW, Lu CW, Huang SK, Wang SJ (2013). Berberine Inhibits the Release of Glutamate in Nerve Terminals from Rat Cerebral Cortex. PLoS One.

[19] Kim M, Shin MS, Lee JM, Cho HS, Kim CJ, Kim YJ, Choi HR, Jeon JW (2014). Inhibitory Effects of Isoquinoline Alkaloid Berberine on Ischemia-Induced Apoptosis via Activation of Phosphoinositide 3-Kinase/Protein Kinase B Signaling Pathway. Int Neurourol J.

[20] Kroemer G, Mariño G, Levine B (2010). Autophagy and the integrated stress response. Mol Cell.

[21] Zhao H, Chen S, Gao K, Zhou Z, Wang C, Shen Z, Guo Y, Li Z, Wan Z, Liu C, Mei X (2017). Resveratrol protects against spinal cord injury by activating autophagy and inhibiting apoptosis mediated by the SIRT1/AMPK signaling pathway. Neuroscience.

[22] Wang C, Liu C, Gao K, Zhao H, Zhou Z, Shen Z, Guo Y, Li Z, Yao T, Mei X (2016). Metformin preconditioning provide neuroprotection through enhancement of autophagy and suppression of inflammation and apoptosis after spinal cord injury. Biochem Biophys Res Commun.

[23] Bai L, Mei X, Shen Z, Bi Y, Yuan Y, Guo Z, Wang H, Zhao H, Zhou Z, Wang C, Zhu K, Li G, Lv G (2017). Netrin-1 Improves Functional Recovery through Autophagy Regulation by Activating the AMPK/mTOR Signaling Pathway in Rats with Spinal Cord Injury. Sci Rep.

[24] Wang J, Qi Q, Feng Z, Zhang X, Huang B, Chen A, Prestegarden L, Li X, Wang J (2016). Berberine induces autophagy in glioblastoma by targeting the AMPK/mTOR/ULK1-pathway. Oncotarget.

[25] Wang H, Li K, Ma L, Wu S, Hu J, Yan H, Jiang J, Li Y (2017). Berberine inhibits enterovirus 71 replication by downregulating the MEK/ERK signaling pathway and autophagy. Virol J.

[26] Zhang Q, Bian H, Guo L, Zhu H (2016). Pharmacologic preconditioning with berberine attenuating ischemia-induced apoptosis and promoting autophagy in neuron. Am J Transl Res.

[27] Tong N, Zhang J, Chen Y, Li Z, Luo Y, Zuo H, Zhao X (2012). Berberine sensitizes mutliple human cancer cells to the anticancer effects of doxorubicin in vitro. Oncol Lett.

[28] Lau CW, Yao XQ, Chen ZY, Ko WH, Huang Y (2001). Cardiovascular actions of berberine. Cardiovasc Drug Rev.

[29] Hsu YY, Chen CS, Wu SN, Jong YJ, Lo YC (2012). Berberine activates Nrf2 nuclear translocation and protects against oxidative damage via a phosphatidylinositol 3-kinase/Akt-dependent mechanism in NSC34 motor neuron-like cells. European Journal of Pharmaceutical Sciences.

[30] Ahmed T, Gilani AU, Abdollahi M, Daglia M, Nabavi SF, Nabavi SM (2015). Berberine and neurodegeneration: A review of literature. Pharmacological Reports : PR.

[31] Keller CW, Lünemann JD (2017). Autophagy and Autophagy-Related Proteins in CNS Autoimmunity. Front Immunol.

[32] Ohsumi Y (2001). Molecular dissection of autophagy: two ubiquitin-like systems. Nat Rev Mol Cell Biol.

[33] Mizushima N, Kuma A, Kobayashi Y, Yamamoto A, Matsubae M, Takao T, Natsume T, Ohsumi Y, Yoshimori T (2003). Mouse Apg16L, a novel WD-repeat protein, targets to the autophagic isolation membrane with the Apg12-Apg5 conjugate. J Cell Sci.

[34] Fujita N, Itoh T, Omori H, Fukuda M, Noda T, Yoshimori T (2008). The Atg16L complex specifies the site of LC3 lipidation for membrane biogenesis in autophagy. Mol Biol Cell.

[35] Guo J, Wang L, Wang L, Qian S, Zhang D (2016). Berberine Protects Human Umbilical Vein Endothelial Cells against LPS-Induced Apoptosis by Blocking JNK-Mediated. Signaling.

[36] Ma X, Jiang Y, Wu A, Chen X, Pi R, Liu M, Liu Y (2010). Berberine attenuates experimental autoimmune encephalomyelitis in C57 BL/6 mice. PLoS One.

[37] Zhou H, Feng L, Xu F, Sun Y, Ma Y, Zhang X, Liu H, Xu G, Wu X, Shen Y, Sun Y, Wu X, Xu Q (2017). Berberine inhibits palmitate-induced NLRP3 inflammasome activation by triggering autophagy in macrophages: A new mechanism linking berberine to insulin resistance improvement. Biomedicine & Pharmacotherapy.

[38] Dinesh P, Rasool M (2017). Berberine, an isoquinoline alkaloid suppresses TXNIP mediated NLRP3 inflammasome activation in MSU crystal stimulated RAW 264.7 macrophages through the upregulation of Nrf2 transcription factor and alleviates MSU crystal induced inflammation in rats. Int Immunopharmacol.

[39] Nadjafi S, Ebrahimi SA, Rahbar-Roshandel N (2014). Protective Effects of Berberine on Oxygen-Glucose Deprivation/Reperfusion on Oligodendrocyte Cell Line (OLN-93). Int J Prev Med.

[40] Cui HS, Matsumoto K, Murakami Y, Hori H, Zhao Q, Obi R (2009). Berberine exerts neuroprotective actions against in vitro ischemia-induced neuronal cell damage in organotypic hippocampal slice cultures: involvement of B-cell lymphoma 2 phosphorylation suppression. Biol Pharm Bull.

[41] Han AM, Heo H, Kwon YK (2012). Berberine promotes axonal regeneration in injured nerves of the peripheral nervous system. J Med Food.

[42] Kilkenny C, Browne WJ, Cuthill IC, Emerson M, Altman DG (2010). Improving bioscience research reporting: the ARRIVE guidelines for reporting animal research. PLoS Biol.

[43] Gao K, Shen Z, Yuan Y, Han D, Song C, Guo Y, Mei X (2016). Simvastatin inhibits neural cell apoptosis and promotes locomotor recovery via activation of Wnt/β-catenin signaling pathway after spinal cord injury. J Neurochem.

[44] Wang H, Wang Y, Li D, Liu Z, Zhao Z, Han D, Yuan Y, Bi J, Mei X (2015). VEGF inhibits the inflammation in spinal cord injury through activation of autophagy. Biochem Biophys Res Commun.

[45] Li D, Wang G, Han D, Bi J, Li C, Wang H, Liu Z, Gao W, Gao K, Yao T, Wan Z, Li H, Mei X (2016). MP Resulting in Autophagic Cell Death of Microglia through Zinc Changes against Spinal Cord Injury. BioMed Research Iinternational.

